# Assessing the Impacts of Climate Change on the Potential Geographical Distribution of *Lycium ruthenicum* in China

**DOI:** 10.3390/biology14101379

**Published:** 2025-10-09

**Authors:** Cheng Li, Yuli Gu, Bo Liu, Kwok Pan Chun, Thanti Octavianti, Mou Leong Tan, Yongping Wu, Lei Zhong

**Affiliations:** 1 Department of Ecology, School of Plant Protection/Jiangsu Province Engineering Research Center of Green Pesticides, Yangzhou University, Yangzhou 225009, China; mz120231474@stu.yzu.edu.cn; 2 College of Hydraulic Science and Engineering, Yangzhou University, Yangzhou 225009, China; boliu@yzu.edu.cn; 3 CATE School of Architecture and Environment, University of the West of England, Bristol BS16 1QY, UK; kwok.chun@uwe.ac.uk (K.P.C.); thanti.octavianti@uwe.ac.uk (T.O.); 4 Geography Section, School of Humanities, Universiti Sains Malaysia, Penang 11800, Malaysia; mouleong@usm.my; 5 College of Physical Science and Technology, Yangzhou University, Yangzhou 225002, China; ypwu@yzu.edu.cn

**Keywords:** climate change, *Lycium ruthenicum*, potential distribution, MaxEnt model, China

## Abstract

**Simple Summary:**

*Lycium ruthenicum* is a perennial thorny shrub belonging to the family Solanaceae and is classified as a second-grade protected plant in China. It has important ecological functions and medicinal value but is threatened by climate change. By optimizing the maximum entropy model, we estimate its current potential habitat range to be approximately 2.25 × 10^6^ km^2^, predominantly distributed in northwestern China. Under future climate scenarios, its suitable habitats will gradually shrink and primarily shift northeastward as climate change progresses. These findings provide scientific support to guide the conservation of *L. ruthenicum* in China.

**Abstract:**

Understanding the climate change impacts on the geographical distribution of plant species is vital for biodiversity conservation. *Lycium ruthenicum*, a second-grade protected plant in China, holds considerable medicinal and ecological value; however, its potential habitat distribution under climate change remains uncertain. By utilizing occurrence records and geographical and environmental data, we optimized a maximum entropy model and evaluated the current and future potential habitat suitability of *L. ruthenicum* in China. The main results were as follows: (1) The distribution of *L. ruthenicum* was primarily influenced by the precipitation of the warmest quarter, topsoil base saturation, precipitation seasonality, precipitation of the coldest quarter, and minimum temperature of the coldest month. (2) Under the current conditions, the potential suitable area of *L. ruthenicum* was approximately 2.25 × 10^6^ km^2^ in China, predominantly distributed in Xinjiang, Qinghai, Gansu, Ningxia, and Inner Mongolia. (3) An obvious reduction in the predicted suitable area of *L. ruthenicum* was found under future climate scenarios, with the centroid primarily shifting northeastward. These findings highlight the potential vulnerability of this medicinally and ecologically important species and underscore the urgent need for targeted conservation strategies to ensure its long-term survival.

## 1. Introduction

With increasing atmospheric greenhouse gas concentrations, global average surface temperature has risen by approximately 1 °C since the pre-industrial era [1,2]. This rise in temperature has resulted in more extreme weather events, which have adversely affected biodiversity and ecosystem stability [3,4,5]. Plants, as a crucial component of terrestrial ecosystems, are particularly sensitive to climate change [6,7]. For example, climate change can alter the habitats on which plants depend, thereby influencing shifts in their original geographical distribution [8,9]. A previous study indicated that many plant species need to shift their ranges by more than 1 km/year to keep pace with climate change [10]. These distribution changes can disrupt existing ecological balances and lead to ecosystem instability [11,12]. Therefore, understanding how climate change affects the potential ranges of plant species is essential for biodiversity conservation and sustainable ecosystem management.

Species distribution models (SDMs) have become an important tool for predicting potential geographical ranges of plant species [13,14]. Commonly used SDM techniques include the maximum entropy (MaxEnt) model [15], genetic algorithm for rule-set prediction (GARP) [16], BIOCLIM program [17], generalized linear model (GLM) [18], and random forest (RF) algorithm [19]. Among these techniques, the MaxEnt model is particularly esteemed for its effectiveness and accuracy [20,21]. It handles complex interactions between species occurrence data and environmental variables, generating visual outputs such as probability distribution maps and variable importance metrics [22,23]. Nowadays, a number of works have been conducted using the MaxEnt model to analyze the environmental responses and potential habitat distributions of different plant species [24,25,26]. However, recent investigations have shown that relying on default parameters in the MaxEnt model can deteriorate the predictive performance [27,28]. In this case, selecting appropriate parameters for modeling is essential when utilizing the MaxEnt model.

*Lycium ruthenicum*, commonly known as Russian box thorn, is a perennial thorny shrub in Solanaceae [29]. It is widely distributed in China, Central Asia, Russia, Mongolia, India, Pakistan, and Afghanistan, even up to Mediterranean areas [30]. This species exhibits unique characteristics, including being light-loving, drought-resistant, and salinity-resistant, making it suitable for growth in desert and saline–alkali regions. It serves essential ecological functions such as windbreak, sand fixation, and soil and water conservation [31]. Meanwhile, *L. ruthenicum* is a medicinal and edible homologous food due to its abundant anthocyanins and other bioactive components, which can remove free radicals in the body and possess antioxidant, anti-fatigue, and immune enhancing effects [32,33]. Research on *L. ruthenicum* has mainly concentrated on its genetics, bioactive components, and functional applications [34]. However, a comprehensive understanding of its potential distribution and habitat suitability has not been reported. Previous studies have indicated that a variety of geographical and environmental factors jointly determine the potential ranges of plant species [24,25,26,35]. Among such factors, bioclimatic factors describing temperature and water-related annual tendencies, seasonality, and extreme climatic conditions have been frequently regarded as primary determinants [36], leading to several climate-based hypotheses on species diversity patterns [37,38,39]. Apart from climatic factors, soil properties also influence the suitable habitats of plant species. For example, unsuitable soil conditions can limit the expansion of suitable habitats for plant species [40]. For *L. ruthenicum* in arid and saline environments, recent field surveys indicated a contraction in its natural distribution, potentially linked to rising temperatures, altered precipitation patterns, and soil degradation [41]. Therefore, for a robust prediction of its distribution, it is crucial to consider the multiple influencing factors during the modeling process.

As one of the primary distribution areas of *L. ruthenicum*, the Chinese government has classified it as one of the national second-grade protected plants, which are species that are not yet facing immediate extinction but still face threats and require protection due to their ecological, economic, or cultural value, and has enacted laws and regulations to strengthen the protection of wild resources [41,42]. Recent studies indicated that the increase in temperature over China since 1900 has exceeded the global mean, highlighting its heightened sensitivity to climate change [43,44]. Given the ongoing climate change, there is still uncertainty regarding its impact on the potential habitat distribution of *L. ruthenicum* in China during different time periods [45,46]. We hypothesize that climate change will alter the potential distribution of this species, resulting in a contraction of its potential suitable habitat and a shift in its distribution centroid. Therefore, the research aims to (1) examine primary factors affecting the distribution of *L. ruthenicum* and (2) reveal the differences in its potential distribution under current and future climate scenarios. These detailed results are important for deepening our understanding of the ecological adaptations and distribution patterns of *L. ruthenicum* in China, thereby offering crucial insights for conservation and sustainable use strategies under climate change.

## 2. Materials and Methods

### 2.1. Data

#### 2.1.1. Occurrence Records of *L. ruthenicum*

**Figure 1:**
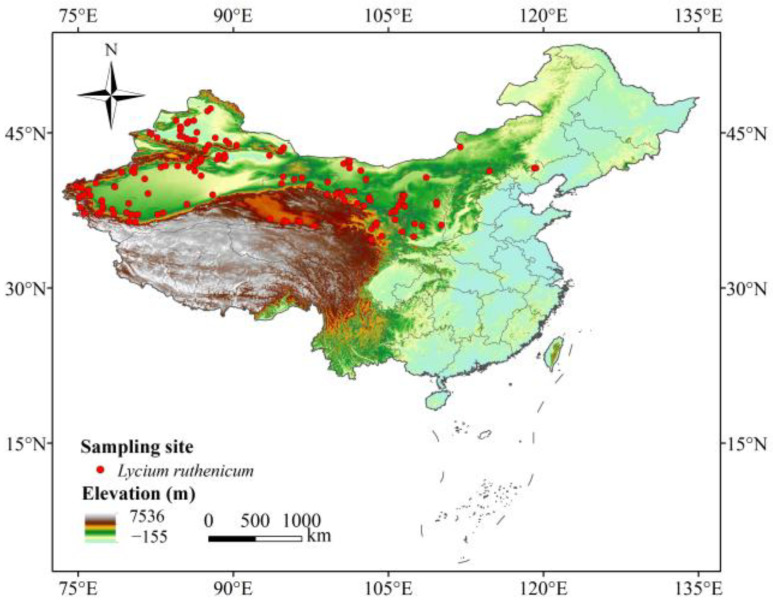
Occurrence records of *L. ruthenicum* in China.

The occurrence records of *L. ruthenicum* were primarily sourced from the Global Biodiversity Information Facility (https://www.gbif.org/, accessed on 10 January 2020) [47], the Chinese Virtual Herbarium (https://www.cvh.ac.cn/, accessed on 10 January 2020), the National Specimen Information Infrastructure of China (http://www.nsii.org.cn/2017/home.php, accessed on 10 January 2020), and the Plant Science Data Center of China (http://www.iplant.cn/, accessed on 10 January 2020). In order to ensure data accuracy and reliability, we first removed duplicate occurrence records and those with missing longitude and latitude coordinates. Furthermore, we utilized the ENMTools program to remove the redundant occurrence data, thereby avoiding the overfitting of prediction results [48]. Finally, there were 161 records of *L. ruthenicum* available for further analysis (Figure 1).

#### 2.1.2. Geographical and Environmental Data

According to previous studies [8,9], we utilized a total of 40 geographical and environmental factors that can affect the distribution of *L. ruthenicum* in our study (Table S1). Among these, 19 bioclimatic variables were derived from https://www.worldclim.org/data/index.html (accessed on 10 January 2020), covering five periods (contemporary, 2030s, 2050s, 2070s, and 2090s) at a 2.5 min resolution. For future climate scenarios, data from shared socioeconomic pathways (SSP126: Low forcing category, radiative forcing reaches 2.6 W/m^2^ in 2100; SSP245: Medium forcing category, radiative forcing reaches 4.5 W/m^2^ in 2100; SSP370: High forcing category, radiative forcing reaches 7.0 W/m^2^ in 2100; and SSP585: High forcing category, radiative forcing reaches 8.5 W/m^2^ in 2100) were from the Beijing Climate Center Climate System Model [49].

In addition to bioclimatic variables, this study incorporated 18 soil properties from the topsoil layer and 3 topographic factors (elevation, slope, and aspect). These data were from the Harmonized World Soil Database and the National Earth System Science Data Center of China, respectively. Furthermore, we resampled the soil and topographic data into a 2.5 min resolution to match the climate data in this study.

### 2.2. Methods

In this study, we first screened suitable geographical and environmental variables according to the percentage contribution of variables and Pearson correlation analysis. Subsequently, we optimized parameters for the MaxEnt model using the R package ENMeval v.2.0.4. Following this, we imported the filtered occurrence records of *L. ruthenicum* and selected variables into the optimized MaxEnt model to predict the potential geographical distribution of *L. ruthenicum* in China. Finally, we conducted statistical analyses on the area of each suitability class and the centroid location of suitable areas for different periods using ArcGIS 10.2 software.

#### 2.2.1. Correlation Analysis of Geographical and Environmental Factors

In order to avoid multicollinearity among geographical and environmental factors, the filtered occurrence records of *L. ruthenicum* along with 40 geographical and environmental factors were loaded into the MaxEnt model, which was calculated 10 times to assess the contribution of each factor [27]. Subsequently, the relationships among these factors were analyzed using Pearson correlation (Figure S1). When the correlation coefficient between two factors was high (|r| > 0.7), only the highest contributing factor was retained [50]. After these steps, a total of 19 factors were screened (Table 1).

#### 2.2.2. Model Construction

In this study, MaxEnt v.3.4.1 was employed to investigate the potential geographical distribution of *L. ruthenicum* in China. According to previous studies [51,52], the model’s performance was significantly affected by the configuration of two parameters, i.e., feature combinations (FCs) and regularization multiplier (RM) [51]. Therefore, it is crucial to optimize these two parameters. Considering the available feature types including Linear (L), Quadratic (Q), Product (P), Threshold (T) and Hinge (H), six FCs were employed (L, LQ, H, LQH, LQHP, and LQHPT). Additionally, eight RM values were set, with a range from 0.5 to 4.0 in steps of 0.5, leading to 48 parameter combinations. The optimal combination was then determined using the R package ENMeval based on the metrics such as the Akaike information criterion correction (AICc) and 10% training omission rate (OR10) [52].

We imported the filtered occurrence records of *L. ruthenicum* along with 19 selected factors into the MaxEnt model, applied the optimal parameter combinations, and repeated the calculation 10 times. In this case, the occurrence records were divided into training and testing sets at a ratio of 75:25, with the number of iterations and background points of 500 and 10,000, respectively [53].

#### 2.2.3. Model Evaluation and Analysis

Model prediction accuracy was assessed using the area under the Receiver Operating Characteristic (ROC) curve (AUC), which varied between 0 and 1. Generally, a higher AUC value indicates better predictive accuracy [54]. For example, an AUC value exceeding 0.9 indicates excellent accuracy.

The prediction results were categorized into four classes of potential suitable habitats using the Jenks method implemented in ArcGIS 10.2 software [55]. These classes included unsuitable area (<0.1), low suitable area (0.1–0.3), medium suitable area (0.3–0.5), and high suitable area (>0.5). Furthermore, the area of each suitability class and the centroid location of suitable areas were calculated for different periods using ArcGIS 10.2 software [56].

## 3. Results

### 3.1. Model Optimization and Evaluation

**Figure 2:**
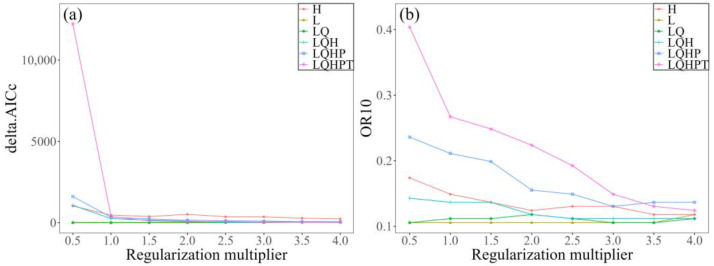
Changes in (**a**) delta.AICc and (**b**) OR10 under different parameter combinations. The delta.AICc quantifies how much a model’s corrected Akaike Information Criterion (AICc) exceeds the lowest AICc found across all parameter combinations, whereas OR10 denotes the 10% training omission rate.

In order to improve model prediction accuracy, a total of 48 parameter combinations of FCs and RMs were evaluated using the ENMeval package. With the default parameters (RM = 1 and FC = LQHPT), the model had a delta.AICc of 348.144 (Figure 2). In contrast, the model utilizing RM = 1 and FC = LQ had the lowest delta.AICc value (i.e., delta.AICc = 0). Furthermore, the OR10 value associated with this parameter combination was much lower than that obtained with the default parameters. Therefore, the optimal parameter configuration was RM = 1 and FC = LQ.

**Figure 3:**
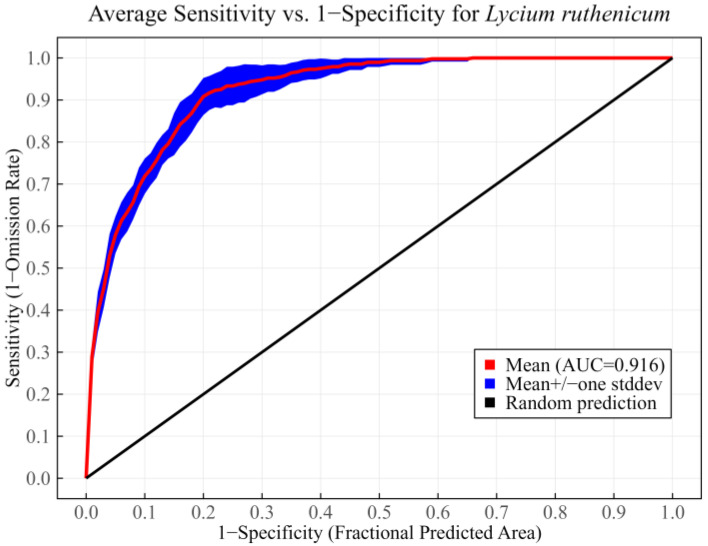
The ROC curve of the optimized MaxEnt model.

The MaxEnt model repeated the calculation 10 times using the optimal parameters, and then the ROC curve was generated (Figure 3). In this study, the AUC was employed to assess predictive accuracy. The average training AUC was 0.946 ± 0.003, while the average testing AUC was 0.916 ± 0.011. These results suggested that the optimized model had excellent predictive accuracy in characterizing potential suitable habitats of *L. ruthenicum*.

### 3.2. Primary Influencing Factors and Response Curve Analysis

The Jackknife test was employed to analyze primary factors affecting the habitat distribution of *L. ruthenicum*. Table 2 shows the percentage contribution of each influencing factor. The ranking of contribution rates showed the top five factors as follows: the precipitation of warmest quarter (Bio18, 46%), topsoil base saturation (T_BS, 11.9%), precipitation seasonality (Bio15, 11.2%), the precipitation of the coldest quarter (Bio19, 8.7%), and the minimum temperature of the coldest month (Bio6, 5.8%). Together, these factors accounted for 83.6% of the total contribution. Furthermore, the cumulative permutation importance of these five factors reached 79.5%. These results suggested that the five factors primarily influenced the distribution pattern of *L. ruthenicum* in China.

**Figure 4:**
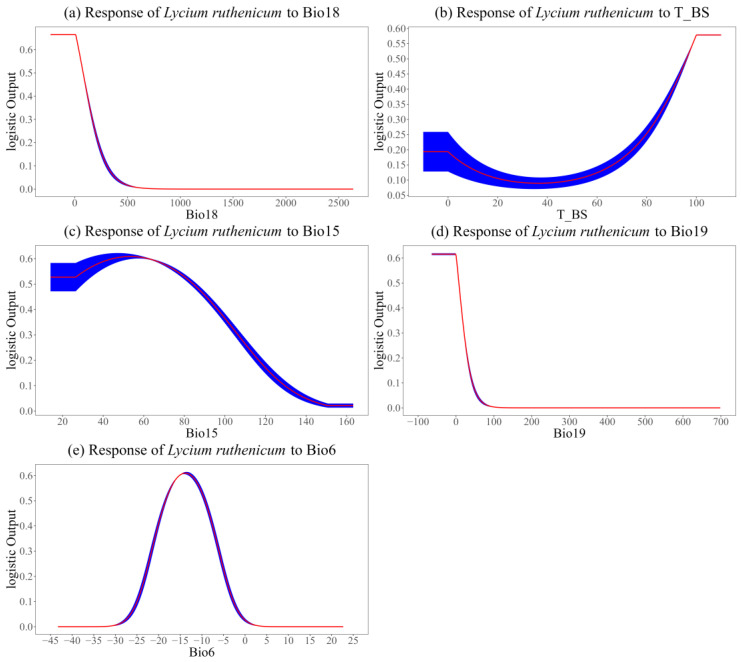
Response curves of primary influencing factors. Red lines and blue areas show the average and standard deviation of 10-fold cross-validation.

Figure 4 shows response curves depicting the occurrence probability of *L. ruthenicum* in relation to the primary influencing factors. Interestingly, each factor showed an obvious preference for specific ranges concerning the occurrence probability of *L. ruthenicum*. A probability threshold of 0.3 was employed to determine the optimal range of primary influences on the suitable habitat of *L. ruthenicum*. As shown in Figure 4, the optimal ranges for each influencing factor were as follows: Bio18 (≤165 mm), T_BS (≥84%), Bio15 (≤105), Bio19 (≤20 mm), and Bio6 (−21.5 to −6 °C).

### 3.3. Potential Geographical Distribution of L. ruthenicum Under Current Conditions

**Figure 5:**
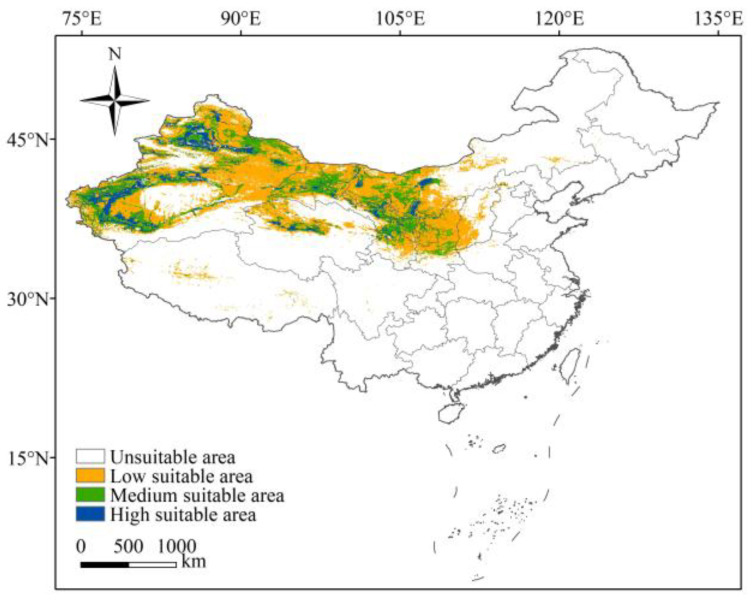
The potential suitable habitats of *L. ruthenicum* in China under the current conditions.

The potential habitat suitability of *L. ruthenicum* in China, under current conditions, were generated using the MaxEnt model, as shown in Figure 5. According to Figure 5, the potential suitable areas of *L. ruthenicum* were distributed in 11 provinces, predominantly in northwestern China. Approximately 0.25 × 10^6^ km^2^ was identified as highly suitable, representing 11.11% of the total suitable area, and these areas were primarily distributed in Xinjiang, Qinghai, Gansu, Ningxia, and Inner Mongolia. Moderately suitable areas covered 0.56 × 10^6^ km^2^, representing 24.89% of the total suitable area, and were typically adjacent to highly suitable areas. In contrast, the lowly suitable area was 1.44 × 10^6^ km^2^, which constituted 64.0% of the total suitable area, and extended into regions such as Shaanxi, Shanxi, Hebei, Henan, Sichuan, and Tibet.

### 3.4. Potential Geographical Distribution of L. ruthenicum Under Future Climate Scenarios

**Figure 6:**
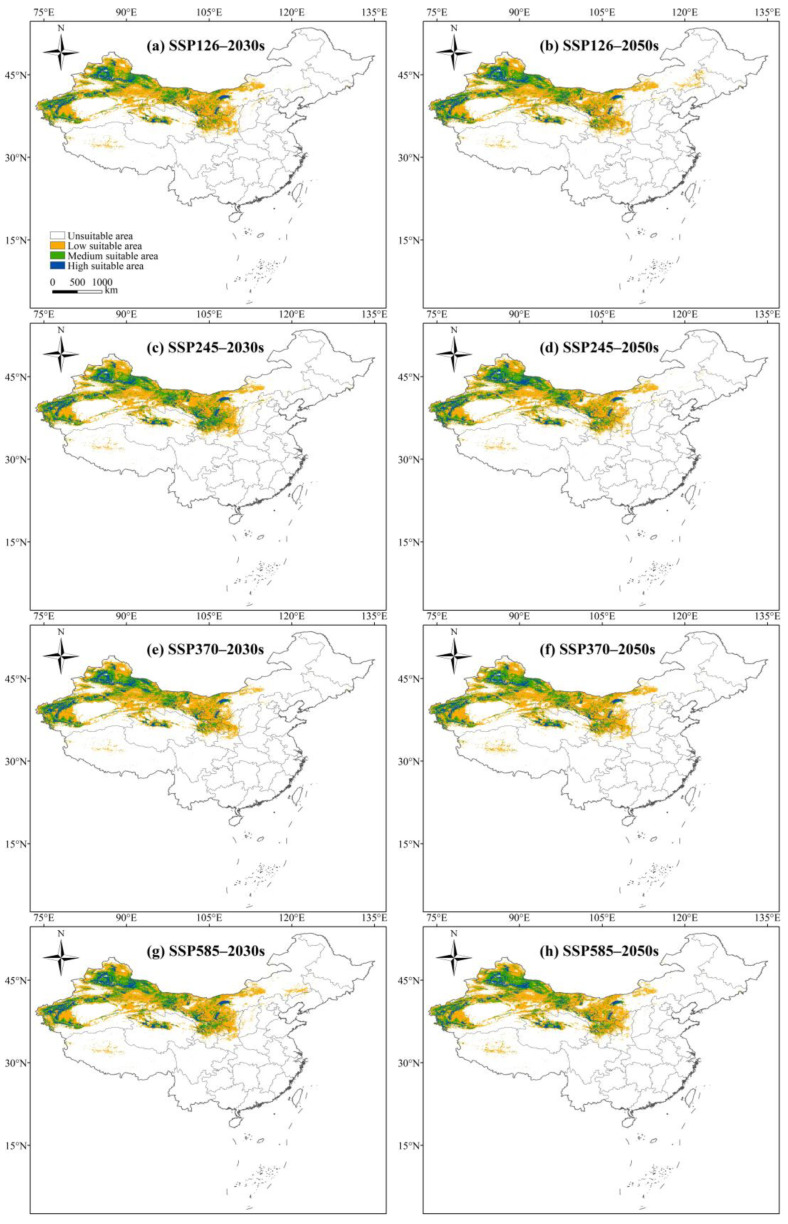
The potential suitable habitats of *L. ruthenicum* in China under future climate scenarios. (**a**) The potential geographical distribution of *L. ruthenicum* under the SSP126 in the 2030s. (**b**) The potential geographical distribution of *L. ruthenicum* under the SSP126 in the 2050s. (**c**) The potential geographical distribution of *L. ruthenicum* under the SSP245 in the 2030s. (**d**) The potential geographical distribution of *L. ruthenicum* under the SSP245 in the 2050s. (**e**) The potential geographical distribution of *L. ruthenicum* under the SSP370 in the 2030s. (**f**) The potential geographical distribution of *L. ruthenicum* under the SSP370 in the 2050s. (**g**) The potential geographical distribution of *L. ruthenicum* under the SSP585 in the 2030s. (**h**) The potential geographical distribution of *L. ruthenicum* under the SSP585 in the 2050s. Different colors in each figure represent different suitability classes, with the meanings of the colors shown in Figure 6a.

The potential habitat suitability of *L. ruthenicum* in China for the 2030s and 2050s was generated using the MaxEnt model based on SSP126, SSP245, SSP370, and SSP585 scenarios, as shown in Figure 6. Given the uncertainty in climate projections, the potential habitat suitability of *L. ruthenicum* for the 2070s and 2090s can be shown in Figure S2. The suitable distributions of *L. ruthenicum* under future climate scenarios showed a general similarity to its distribution under current conditions. Highly suitable areas predominantly remained in Xinjiang, Qinghai, Gansu, Ningxia, and Inner Mongolia, while suitable areas were relatively limited in Shanxi, Hebei, and Tibet. However, the area within each suitability class of *L. ruthenicum* exhibited varying degrees of change over time (Table 3 and Table S2). For example, the area of highly suitable habitats remained relatively stable, whereas moderate and low suitability areas decreased, resulting in an expansion of unsuitable areas of *L. ruthenicum* compared to contemporary levels.

The potential suitable habitats of *L. ruthenicum* showed obvious differences under future climate scenarios (Table 1 and Table S2). However, the overall trend indicated a shrinking distribution range compared to contemporary levels. High suitability habitats peaked at 0.28 × 10^6^ km^2^ in the 2030s and 2050s under the SSP245 scenario but declined to 0.20 × 10^6^ km^2^ by the 2090s under the SSP585 scenario, with a 20% reduction from contemporary levels. Moderately suitable habitats generally decreased over time, with the exception of the 2030s, under the SSP245 scenario. The most substantial decline in moderately suitable areas occurred in the 2090s under the SSP585 scenario, with a 21.4% reduction relative to contemporary levels. Furthermore, the area of low suitability habitats experienced the greatest decrease in the 2070s under the SSP585 scenario, with a 20.8% reduction compared to contemporary levels, although its area showed a consistent downward trend over time.

### 3.5. Centroid Shifts in the Potential Distribution of L. ruthenicum Under Climate Change

**Figure 7:**
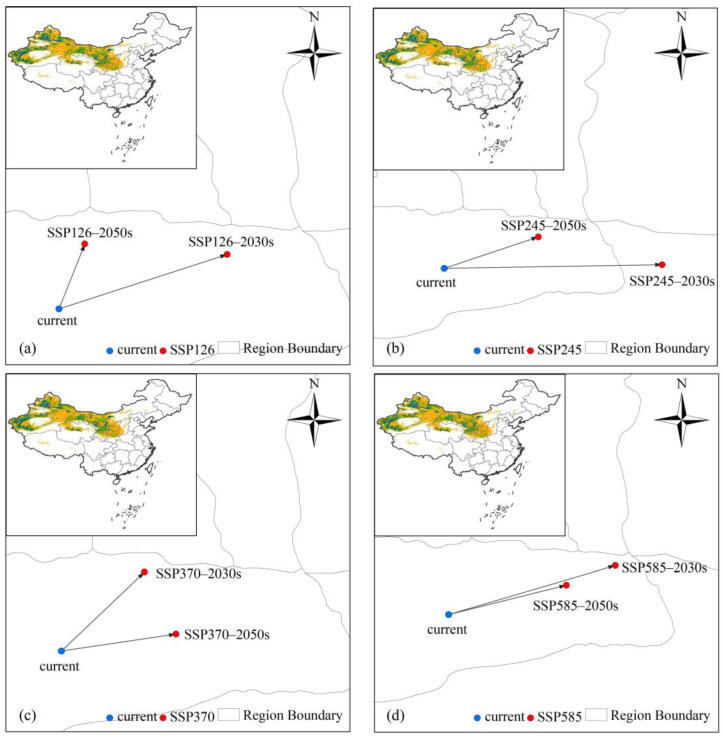
The centroid migration of *L. ruthenicum* under future climate scenarios. (**a**) The centroid migration in potential suitable areas of *L. ruthenicum* under the SSP126 in the 2030s and 2050s. (**b**) The centroid migration in potential suitable areas of *L. ruthenicum* under the SSP245 in the 2030s and 2050s. (**c**) The centroid migration in potential suitable areas of *L. ruthenicum* under the SSP370 in the 2030s and 2050s. (**d**) The centroid migration in potential suitable areas of *L. ruthenicum* under the SSP585 in the 2030s and 2050s. The inset in the upper left corner of each figure represents the potential suitable habitats of *L. ruthenicum* in China under the current conditions, which is derived from Figure 5.

The geographical centroid of potential suitable habitats of *L. ruthenicum*, calculated by importing the model output into ArcGIS (Figure 7a–d and Figure S3), exhibited distinct shifts in different periods. Under the current conditions, the centroid coordinates of potential suitable habitats were recorded at 88.1553° E, 40.8586° N. Under future climate scenarios, the centroid of suitable habitats predominantly shifted northeastward over time compared to contemporary levels, with the exception of the 2090s, under the SSP370 and SSP585 scenarios. During the 2090s, migration of the centroid consistently showed a southwestward trend under the SSP370 and SSP585 scenarios (Figure S3). The furthest migration of the centroid reached 185 km in the 2030s under the SSP245 scenario, while the closest migration was recorded at 45 km in the 2050s under the SSP126 scenario.

## 4. Discussion

### 4.1. Primary Factors Affecting the Potential Distribution of L. ruthenicum

According to the correlation analysis combined with the contribution rate of various geographical and environmental factors, we identified Bio18, T_BS, Bio15, Bio19, and Bio6 as the top five factors affecting the distribution of *L. ruthenicum*. Among these, Bio18, Bio15, and Bio19 were precipitation-related factors, underscoring a certain role of precipitation in reflecting the geographical distribution of *L. ruthenicum*. This is largely because *L. ruthenicum* is predominantly found in the arid desert and saline–alkali regions of northwestern China, where precipitation is extremely scarce [30,32]. It should be noted that the response curves between precipitation-related variables and the probability of species presence are likely to reflect a statistical association rather than a biologically causative relationship [52]. In addition to precipitation, temperature is also an important determinant for many plant species [57,58]. A recent study found that the semi-lethal temperatures of *L. ruthenicum* range from −35 to −26 °C [59], suggesting that it can thrive within the optimal range of −21.5 to −6 °C for Bio6.

In desert regions with saline and alkaline soils, soil characteristics are often one of the primary factors determining the distribution of *L. ruthenicum* [41]. We selected several soil factors for modeling; however, only T_BS contributed more than 5%. The result is consistent with the biological characteristics of *L. ruthenicum*, which possesses a well-developed root system and demonstrates adaptability to poor soil conditions, as well as tolerance to high temperatures, nutrient-poor environments, and drought [60]. Some studies pointed out that *L. ruthenicum* is likely adapted to deep rooting and reliance on groundwater rather than direct rainfall [61,62]. This adaptation is key to its survival in arid environments. In fact, these precipitation-related variables do not denote a direct physiological reliance on rainfall; rather, they serve as highly effective proxy indicators for the complex abiotic conditions that define its preferred habitat [35]. Therefore, the model can be used to identify the arid environmental regime that results in the specific soil conditions required for this species. The strong performance of these precipitation-related variables likely reflects their utility in mapping this broader abiotic niche. We acknowledge that this is an indirect approach and that the ideal model would incorporate direct data on groundwater depth and salinity. However, such datasets are currently unavailable. In their absence, bioclimatic variables provide a powerful and widely used proxy indicator [63].

While this study focused on the potential distribution of *L. ruthenicum* in China and identified its primary influencing factors, this species also occurs in other regions, such as Central Asia, Russia, Mongolia, India, Pakistan, and Afghanistan, even up to Mediterranean areas [30]. The scarcity of reliably georeferenced occurrence records in these regions precludes their inclusion in the MaxEnt model [64]. Nevertheless, the primary factors identified in China and their optimal ranges provide critical baseline parameters for preliminary habitat screening in adjacent regions. Notably, future precipitation projections exhibit complex spatial patterns that diverge from the consistent global warming trend, introducing uncertainty regarding the future potential distribution of *L. ruthenicum* in regions outside of China. Therefore, future transnational collaborations to compile verified occurrence records and standardized environmental datasets will be essential for evaluating the potential habitat suitability of *L. ruthenicum* across a broad geographic range.

### 4.2. Changes in the Potential Suitable Habitats of L. ruthenicum

Our findings indicate that the current potential habitat range of *L. ruthenicum* is approximately 2.25 × 10^6^ km^2^, consistent with previous estimates that place its range primarily between 1.11 × 10^6^ and 2.84 × 10^6^ km^2^ [41,42,45,46]. The discrepancies appear to stem from different input variables and the optimized MaxEnt model. On the other hand, the current potential suitable habitats of *L. ruthenicum* were predominantly found in Xinjiang, Qinghai, Gansu, Ningxia, and Inner Mongolia, consistent with the distribution of occurrence records [30,32]. These regions lie in northwestern China and share similar climatic characteristics [65]. Under future climate scenarios, the predicted suitable areas of *L. ruthenicum* varied in different periods and scenarios, yet the overall trend pointed to a contraction. This pattern mirrors those reported for other desert plants in northwestern China [66] and is attributable to several ecological mechanisms driven by changing hydrothermal conditions. First, the projected increase in mean temperature and frequency of heatwaves may exceed the species’ photosynthetic optimum [67], causing thermal stress and cellular dysfunction, while simultaneously exacerbating water loss through transpiration [68]. Second, although *L. ruthenicum* relies on groundwater, the replenishment of this resource is intrinsically linked to precipitation [69]. Projected increases in precipitation seasonality and the frequency of severe droughts threaten to lower groundwater tables and intensify soil moisture deficits, potentially pushing the species beyond its hydraulic safety margin in some parts of its current range [70]. Moreover, the decline in suitable areas of *L. ruthenicum* was non-linear, underscoring the complex effects of climate change on habitat suitability [71]. Consistent with broader evidence [72,73], our results indicated that the centroid of suitable habitats of *L. ruthenicum* shifted predominantly northeastward under future climate conditions compared to the present. These distribution changes represent an adaptive response to climate warming [74], compelling *L. ruthenicum* to relocate to newly suitable areas.

Although our study predicted the potential distribution of *L. ruthenicum* under climate change, its realized distribution typically constitutes only a subset of the potential range due to intensive anthropogenic pressures [75]. For example, over-harvesting in potentially suitable areas directly threatens the growth and reproductive success of *L. ruthenicum* [76], while land conversion and habitat fragmentation disrupt habitat connectivity and hinder the establishment and persistence of *L. ruthenicum* populations [77]. As a second-grade protected plant in China, *L. ruthenicum* is not facing immediate extinction, but it remains under threat and requires active protection [41]. Furthermore, the Chinese government has enacted laws and regulations to restrict large-scale or destructive harvesting and to strengthen the protection of wild resources [42]. In order to reduce the negative impacts of anthropogenic pressures and better protect *L. ruthenicum*, we recommend integrated conservation strategies that include regulating harvests through quotas, monitoring potential suitable habitats, protecting connectivity corridors, and establishing climate-adapted cultivation in newly suitable areas, thereby maximizing the economic and ecological value of *L. ruthenicum*.

### 4.3. Limitations of the Study

This study faced limitations, primarily from three aspects. First, the issue of layer resolution was a shortfall of this study. According to the previous studies [27,78], the 2.5 min resolution of environmental factors was suitable for China as the study region. This suggested that the soil and topographic data were resampled into a 2.5 min resolution to match the climate data utilized, which could introduce uncertainties during the resampling process and affect the prediction results. Second, soil variables were held constant for both current and future modeling processes due to the lack of future datasets on soil properties [55]. We acknowledge that this assumption limits the validity of our long-term predictions. Our predictions should therefore be interpreted as the impact of climate change on the potential geographical distribution of *L. ruthenicum* assuming that current soil conditions remain unchanged. Third, some studies emphasized that biotic interactions may also matter at macroecological scales, and these interactions are likely to play a role in shaping the dynamic responses of species to changes in climate [79,80]. However, research on such processes on *L. ruthenicum* remains limited. Available findings include the following: (1) salt-tolerant rhizosphere bacteria can enhance plant growth through nitrogen fixation, phosphorus solubilization, indole-3-acetic acid production, and siderophore synthesis [81]; (2) tissue-specific endophytic fungi-colonizing root, stem, leaf, and fruit can modulate drought and disease resistance, and may produce secondary metabolites that feedback on host fitness [82]; (3) at Qingtu Lake, the terminal reach of the Shiyang River, *L. ruthenicum* exhibits high niche overlap with *Peganum harmala* L., suggesting potential interspecific competition [83]; (4) *L. ruthenicum* relies on insects for pollination and on birds and rodents for seed dispersal, thereby forming the mutualistic interactions [31]; (5) climate-driven increases in pest outbreaks impose additional stress on *L. ruthenicum* [84]. Despite these insights, fine-scale experimental datasets are still scarce and how local interactions affect broad-scale species distributions is still uncertain [80]; consequently, omitting these biotic drivers in the MaxEnt model may overestimate habitat suitability in regions experiencing inhibitory interactions. With the continuous development of big data and artificial intelligence technology [85], future work should incorporate biotic interactions and anthropogenic impacts and integrate updated environmental datasets and hybrid SDMs with dynamic models to elucidate the potential and realized distributions of *L. ruthenicum* at finer scales and across broader geographic ranges.

## 5. Conclusions

We evaluated the current and future potential habitat distribution of *L. ruthenicum* based on the multi-source data and an optimized MaxEnt model. By integrating variables related to temperature, precipitation, soil, and topography, five primary factors influencing habitat suitability were identified, including the Bio18 (46%), T_BS (11.9%), Bio15 (11.2%), Bio19 (8.7%), and Bio6 (5.8%). At present, potential suitable habitats were predominantly found in northwestern China, covering an area of 2.25 × 10^6^ km^2^. Future climate projections indicated a general reduction in suitable habitats for *L. ruthenicum*, with the most significant decrease expected in the 2090s under the SSP585 scenario. These results can provide valuable insights to develop specific conservation and sustainable use strategies for climate change impacts on *L. ruthenicum* in China.

## Figures and Tables

**Table 1:** Geographical and environmental factors used for modeling after screening.

No.	Variable	Description
1	Bio2	Mean diurnal range
2	Bio6	Minimum temperature for coldest month
3	Bio15	Precipitation seasonality
4	Bio18	Precipitation of warmest quarter
5	Bio19	Precipitation of coldest quarter
6	SRTM_DEM	DEM
7	SRTM_SLP	Slope
8	SRTM_ASP	Aspect
9	AWC_CLASS	Available water storage capacity
10	T_BS	Topsoil base saturation
11	T_CACO_3_	Topsoil calcium carbonate
12	T_CEC_CLAY	Cation exchange capacity of the clay fraction in the topsoil
13	T_CEC_SOIL	Cation exchange capacity of the clay fraction in the subsoil
14	T_ECE	Topsoil salinity
15	T_GRAVEL	Topsoil gravel content
16	T_OC	Topsoil organic carbon
17	T_SILT	Topsoil silt fraction
18	T_TEB	Total exchangeable bases in the topsoil
19	T_USDA_TEX	Topsoil texture classification

**Table 2:** The contribution rate of each influencing factor of *L. ruthenicum*.

No.	Variable	Percent Contribution/%	Permutation Importance/%
1	Bio18	46	28
2	T_BS	11.9	5.3
3	Bio15	11.2	9.5
4	Bio19	8.7	30.1
5	Bio6	5.8	6.6
6	AWC_CLASS	3.2	1.2
7	T_USDA_TEX	2.4	3
8	Bio2	2.1	1.4
9	T_CEC_CLAY	1.6	1
10	SRTM_DEM	1.4	2.7
11	T_CEC_SOIL	1.2	4.3
12	T_CACO_3_	1.1	1.1
13	T_SILT	1	3.6
14	T_ECE	0.8	0.3
15	T_OC	0.7	0.8
16	T_TEB	0.5	0.6
17	SRTM_SLP	0.2	0.2
18	SRTM_ASP	0.1	0.1
19	T_GRAVEL	0.1	0.1

**Table 3:** The potential suitable area of *L. ruthenicum* in China under different periods.

Period	Climate Scenarios	No Suitable Area (×10^6^ km^2^)	Low Suitable Area (×10^6^ km^2^)	Medium Suitable Area (×10^6^ km^2^)	High Suitable Area (×10^6^ km^2^)
Current	—	7.36	1.44	0.56	0.25
2030s	SSP126	7.67	1.22	0.48	0.23
	SSP245	7.58	1.17	0.58	0.28
	SSP370	7.69	1.15	0.51	0.26
	SSP585	7.60	1.20	0.54	0.27
2050s	SSP126	7.64	1.19	0.52	0.26
	SSP245	7.67	1.14	0.52	0.28
	SSP370	7.61	1.24	0.52	0.25
	SSP585	7.69	1.17	0.50	0.24

## Data Availability

The data presented in this study are available on request from the corresponding author.

## Supplementary Materials

The following supporting information can be downloaded at: https://www.mdpi.com/article/10.3390/biology14101379/s1, Figure S1: Correlation diagram of geographical and environmental factors for *L. ruthenicum*; Figure S2: The potential suitable habitats of *L. ruthenicum* in the 2070s and 2090s under future climate scenarios; Figure S3: The centroid migration of *L. ruthenicum* in the 2070s and 2090s under future climate scenarios; Table S1: Geographical and environmental factors initially selected in this study; Table S2: The potential suitable area of *L. ruthenicum* in China in the 2070s and 2090s under future climate scenarios.
