# Supplementary material for: Assessing the Impacts of Climate Change on the Potential Geographical Distribution of Lycium ruthenicum in China
Source: Biology (Basel). 2025 Oct 9;14(10):1379. doi: 10.3390/biology14101379 (PMC12562220; doi:10.3390/biology14101379)
Supplement: Supplementary file 1 [file biology-14-01379-s001.zip › biology-3824111-supplementary.pdf]

Supplemental Materials:

Table S1 Geographic and environmental factors initially selected in this study.

No.	Variable	Description
1	Bio1	Annual mean temperature
2	Bio2	Mean diurnal range
3	Bio3	Isothermality
4	Bio4	Temperature seasonality
5	Bio5	Maximum temperature for warmest month
6	Bio6	Minimum temperature for coldest month
7	Bio7	Temperature annual range
8	Bio8	Mean temperature of wettest quarter
9	Bio9	Mean temperature of driest quarter
10	Bio10	Mean temperature of warmest quarter
11	Bio11	Mean temperature of coldest quarter
12	Bio12	Annual precipitation
13	Bio13	Precipitation of wettest month
14	Bio14	Precipitation of driest month
15	Bio15	Precipitation seasonality
16	Bio16	Precipitation of wettest quarter
17	Bio17	Precipitation of driest quarter
18	Bio18	Precipitation of warmest quarter
19	Bio19	Precipitation of coldest quarter
20	SRTM_DEM	DEM
21	SRTM_SLP	Slope
22	SRTM_ASP	Aspect
23	AWC_CLASS	Available water storage capacity
24	T_BS	Topsoil base saturation
25	T_BULK_DEN	Topsoil bulk density
26	T_CACO_3	Topsoil calcium carbonate
27	T_CASO_4	Topsoil gypsum
28	T_CEC_CLAY	Cation exchange capacity of the clay fraction in the topsoil
29	T_CEC_SOIL	Cation exchange capacity of the clay fraction in the subsoil
30	T_CLAY	Topsoil clay fraction
31	T_ECE	Topsoil salinity
32	T_ESP	Topsoil sodicity
33	T_GRAVEL	Topsoil gravel content
34	T_OC	Topsoil organic carbon
35	$T\_PH\_H_2O$	Topsoil pH (H_2O)
36	T_REF_BULK	Topsoil reference bulk density
37	T_SAND	Topsoil sand fraction
38	T_SILT	Topsoil silt fraction
39	T_TEB	Total exchangeable bases in the topsoil
40	T_USDA_TEX	Topsoil texture classification

Table S2 The potential suitable area of *L. ruthenicum* in China in the 2070s and 2090s under future climate scenarios.

Period	Climate Scenarios	No Suitable Area (×10⁶ km²)	Low Suitable Area (×10⁶ km²)	Medium Suitable Area (×10⁶ km²)	High Suitable Area (×10⁶ km²)
Current	—	7.36	1.44	0.56	0.25
2070s	SSP126	7.71	1.17	0.49	0.23
	SSP245	7.67	1.21	0.49	0.24
	SSP370	7.65	1.25	0.48	0.23
	SSP585	7.74	1.19	0.46	0.21
2090s	SSP126	7.69	1.18	0.50	0.24
	SSP245	7.72	1.19	0.47	0.22
	SSP370	7.75	1.19	0.45	0.21
	SSP585	7.81	1.15	0.44	0.20

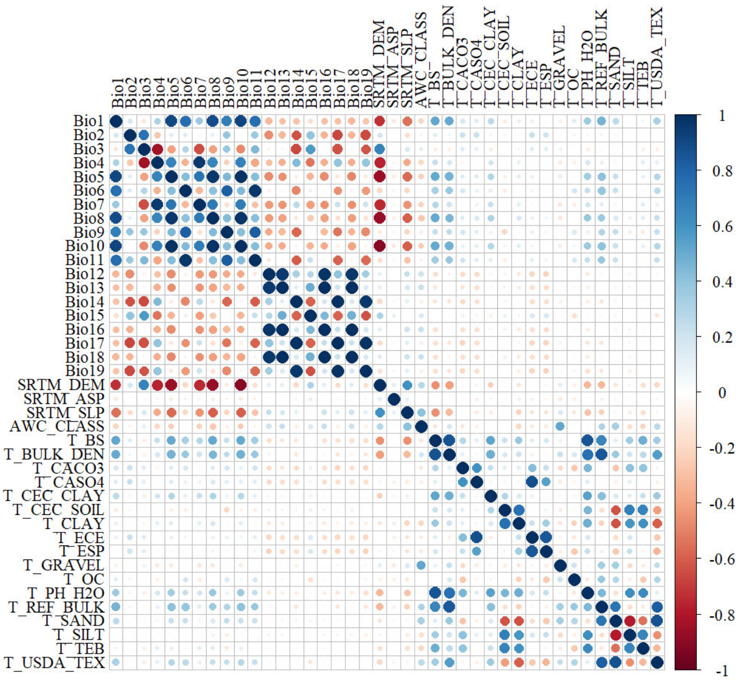

Figure S1 Correlation diagram of geographic and environmental factors for *L. ruthenicum*.

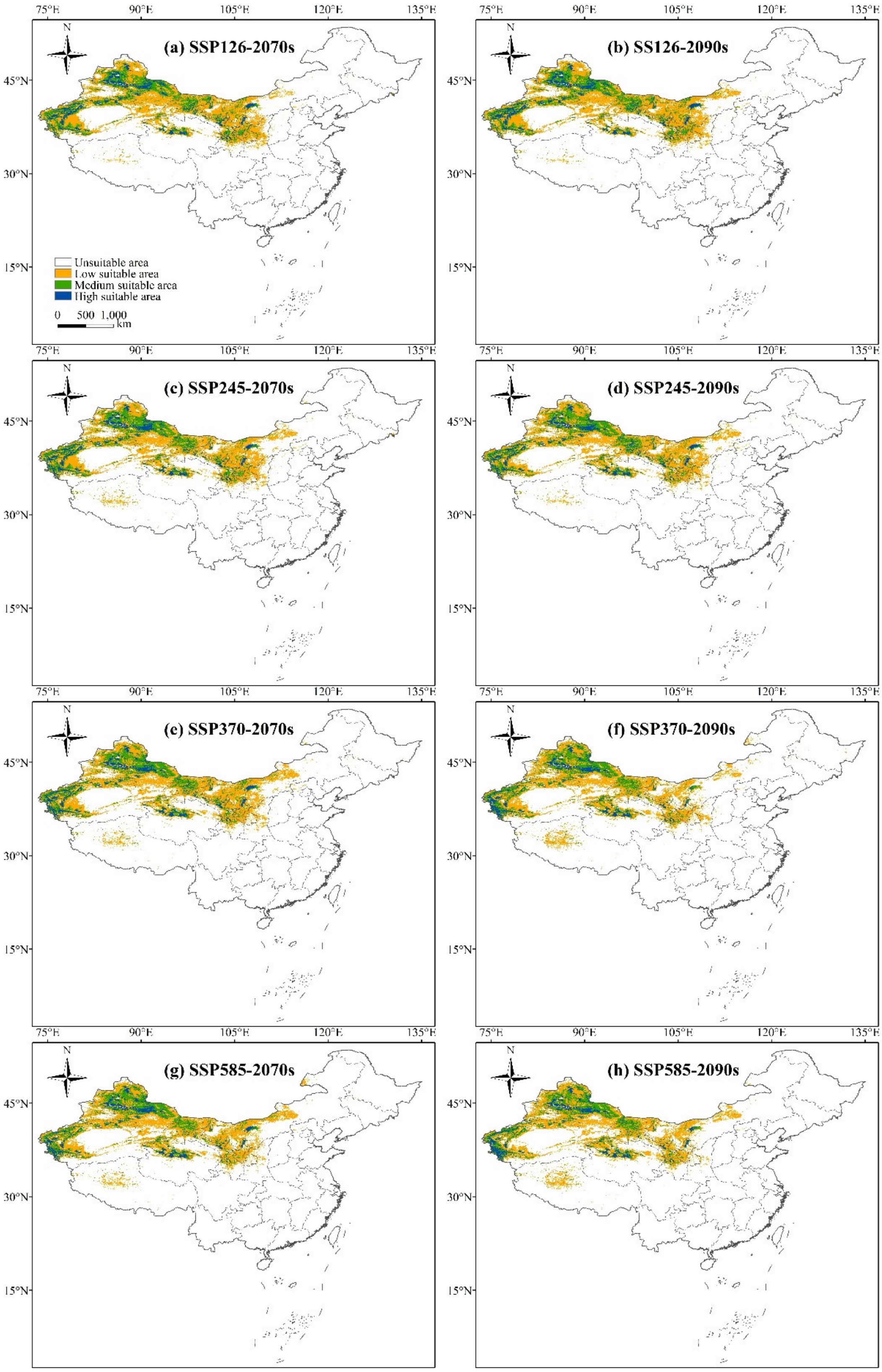

Figure S2 The potential suitable habitats of *L. ruthenicum* in the 2070s and 2090s under future climate scenarios.

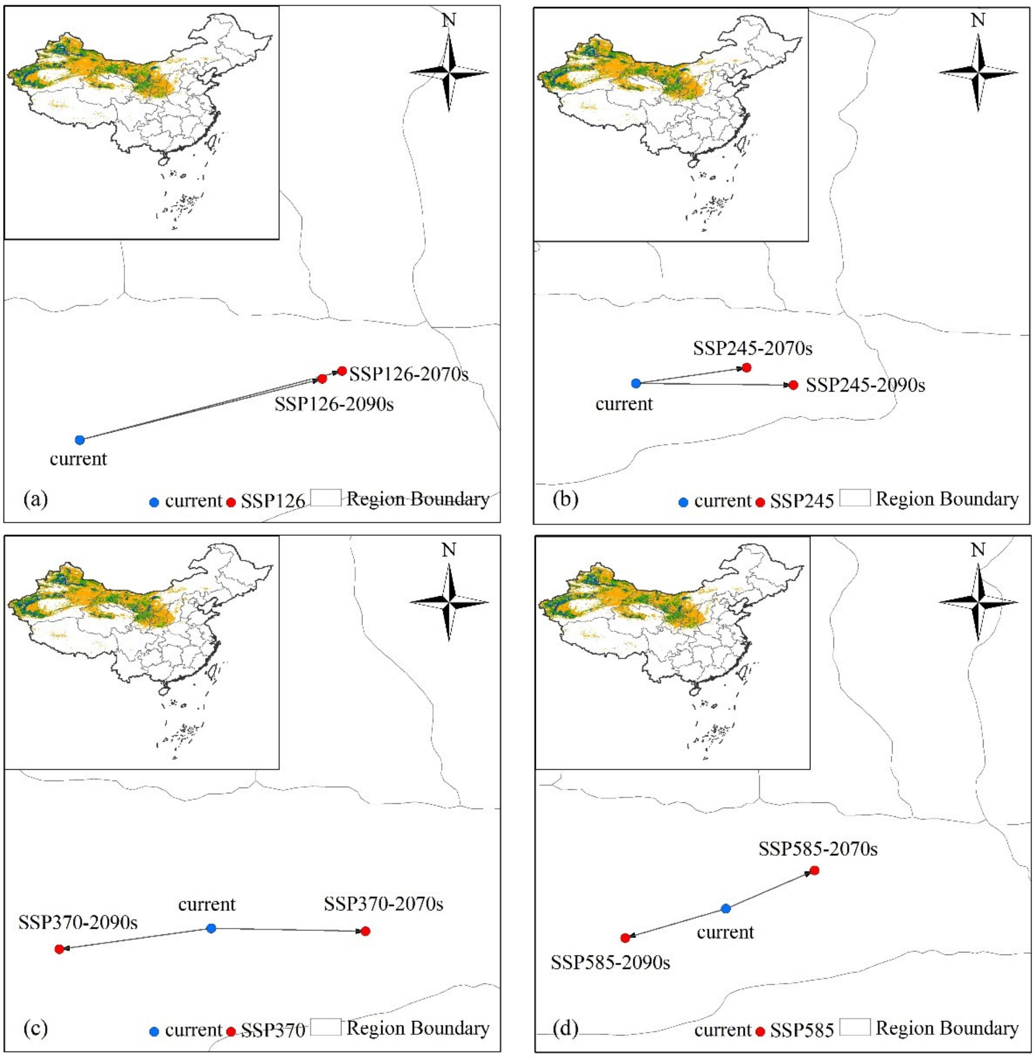

Figure S3 The centroid migration of *L. ruthenicum* in the 2070s and 2090s under future climate scenarios.
